# The Epidemiologic Characteristics of Malignant Mesothelioma Cases in Korea: Findings of the Asbestos Injury Relief System from 2011–2015

**DOI:** 10.3390/ijerph181910007

**Published:** 2021-09-23

**Authors:** Soon-Chan Kwon, Sung-Soo Lee, Min-Sung Kang, Da-An Huh, Yong-Jin Lee

**Affiliations:** 1 Department of Occupational and Environmental Medicine, Soonchunhyang University Cheonan Hospital, Cheonan-si 31151, Korea; sckwon@sch.ac.kr; 2 Department of Preventive Medicine, Soonchunhyang University College of Medicine, Cheonan-si 31151, Korea; sungsool@sch.ac.kr; 3 Asbestos Environmental Health Center, Soonchunhyang University Cheonan Hospital, Cheonan-si 31151, Korea; kms83korea03@hanmail.net; 4 Institute of Health Sciences, Korea University, Seongbuk-gu, Seoul 02841, Korea; black1388@korea.ac.kr

**Keywords:** malignant mesothelioma, asbestos, latent period, survival, incidence

## Abstract

(1) Background: The purpose of this study was to investigate the epidemiological characteristics of malignant mesothelioma in Korea by investigating cases compensated under the asbestos injury relief system. (2) Methods: A total of 407 compensated cases between 2011 and 2015 were reviewed using medical records and resident registrations in order to investigate the dates of diagnosis and death. Asbestos exposure and patients’ general characteristics were investigated through face-to-face interviews. The standardized incidence ratio was calculated as the number of observations from 2005 to 2014 per exposure region in Korea, using the mid-annual population of each region in 2009 as the standard population. (3) Results: Among the 407 cases, 65.1% were male. The pleura and peritoneum were affected in 76.9% and 23.1% of cases, respectively. For peritoneal mesothelioma, the median survival duration was longer (*p* = 0.005), and the proportion of affected women was higher than that in pleural mesothelioma. The standardized incidence ratio (95% CI) by province of primary exposure was Chungnam 3.33 (2.51–4.35), Ulsan 1.85 (0.97–3.21), and Seoul 1.32 (1.06–1.63). (4) Conclusions: Although the representativeness of the data is limited, it is sufficient to assume the epidemiologic characteristics of malignant mesothelioma, help improve the compensation system, and contribute to future policies.

## 1. Introduction

Asbestos is a naturally occurring mineral that has strong heat resistance, durability, insulation, and heat retention, and is also inexpensive. After first being used as a cremation cloth in 450 BC [1], humanity began to use heat-resistant asbestos in earnest with the Industrial Revolution and the two world wars. In the early 20th century, lightweight and heat-resistant materials, such as asbestos, were introduced and widely used for construction [2]. However, asbestos is known to have various adverse health effects. The first case of pulmonary fibrosis due to asbestos was reported in 1924 [3], and was named asbestosis in 1925 [4]. Under the World Health Organization (WHO), the International Agency for Research on Cancer (IARC) found that the risk of lung cancer and malignant mesothelioma in workers exposed to crocidolite and amosite [5], as well as in workers exposed to crocidolite, amosite, and chrysotile [6], increased in 1973 and 1977, respectively. In 2012, all types of asbestos exposure were designated as group 1 carcinogens of lung cancer, malignant mesothelioma, laryngeal cancer, and ovarian cancer. Malignant mesothelioma is a malignant tumor that develops on serous surfaces, such as the pleura or peritoneum [7]. The disease progresses rapidly and the subject dies within 4–12 months of its onset with only supportive care [8]. It is known to occur after exposure to a smaller amount of asbestos as compared to larger amounts that trigger lung cancer [9]. However, similar to other asbestos-related diseases, there is a significant time delay between exposure and disease onset. The latency period is typically longer than 30 years but has also been described as approximately 15 years [10].

In the past, asbestos was used most often in the United States, Japan, and Europe. However, as health hazards caused by exposure to asbestos were discovered, most of these countries banned the use of asbestos in the 1990s and 2000s. Asbestos is now being used by emerging industrial countries such as China, India, Russia, Brazil, and Indonesia, which are still experiencing rapid economic growth. Currently, asbestos is mainly produced in Russia, South Africa, and China [11]. In 1990, the Occupational Safety and Health Act of Korea stipulated that asbestos could be manufactured or used only when licensed. In 1997, Korea banned the import, manufacture, transfer, or use of the most dangerous type of asbestos, crocidolite. In 2009, Korea banned the manufacture and use of asbestos products, except for those that do not have alternative replacements [12]. Kwak et al. (2021) have forecast that the incidence of malignant mesothelioma in Korea will increase until around 2040 using the age–period–cohort model and a Poisson regression model based on asbestos consumption [13].

In Korea, there are few statistics on the epidemiological characteristics of malignant mesothelioma, such as mortality data, cancer registration data, and industrial accident compensation data [14]. Meanwhile, since 2011, a relief system has been provided for people who have been exposed to asbestos and suffered from asbestos-related diseases (primary malignant mesothelioma, primary lung cancer, asbestosis, and diffuse pleural thickening). However, no research has been published on the epidemiologic analysis of malignant mesothelioma in Korea based on the data collected by the asbestos injury relief system. The purpose of this study was to describe and analyze the magnitude and characteristics of malignant mesothelioma caused by asbestos exposure, recognized using the relief system of asbestos injury in Korea.

## 2. Materials and Methods

Between 2011 and 2015, 728 patients or their bereaved families were compensated for asbestos injuries or provided special bereaved recognition under the Asbestos Injury Relief Act. Of these, 321 (43.5%) were unable to complete the survey due to a change of contact information for 180 (24.7%) patients and refusal to participate for 141 (19.4%) patients. In 2015 and 2016, asbestos-related experts (including occupational and environmental medicine specialists and preventive medicine specialists) investigated the general and exposure-related characteristics of those who applied for asbestos injury relief or their bereaved families using structured questionnaires. In addition, with the consent of the participants, the medical and resident registration records were reviewed to investigate the dates of diagnosis and death, as well as the methods of diagnosis and treatment. The survival duration was calculated from the time of diagnosis to the time of death, or in the case of survival, from the time of diagnosis to the time of the investigation in March 2019. The latency period was defined as the period between the estimated initial exposure to asbestos and the definitive diagnosis based on the histology. During the study period, 407 patients were identified. A total of 406 cases were subjected to biopsy analyzed through immunohistochemical staining methods, and radiological diagnosis was used in one case where a specimen could not be obtained due to the patient’s death. The study variables were sex (male vs. female), age at diagnosis (classification: <40, 40–49, 50–59, 60–69, and ≥70 years), smoking history (yes or no), site of onset (pleural versus peritoneal), and occupational exposure to asbestos (yes or no). 

We used independent *t*-tests, the chi-squared method, and Fisher’s exact test to determine the differences in the general and exposure-related characteristics between peritoneal and pleural mesothelioma and between men and women. The primary exposure area was defined as the province that contributed the most to the occurrence of malignant mesothelioma owing to exposure (occupational, environmental, or domestic) at least 15 years before diagnosis. This was determined considering the latency period of malignant mesothelioma, referring to the probability and intensity of environmental and domestic exposure to asbestos by C Magnani et al. (2000) [15]. The standardized incidence ratio (SIR) was calculated as the number of observations from 2005 to 2014 (since, in these years, more than 20 cases per year were consistently diagnosed) by the province of primary exposure, using the mid-annual population of each province of Korea in 2009 as the standard population. Statistical analyses were performed using IBM SPSS statistics software (version 22.0; IBM, New York, NY, USA), and the significance level was set at *p* < 0.05. All participants provided written informed consent, and the Institutional Review Board of our hospital approved the study (IRB: Schca2009-04-001).

## 3. Results

A total of 407 subjects responded during the study period. The mean age ± standard deviation (SD) at the time of diagnosis was 62.49 ± 12.26 years, and there was no significant difference between male (63.09 ± 11.83) and female (61.38 ± 13.00) participants. Men accounted for 65.1% of the participants (*n* = 265). In terms of their ages, 32.9% were ≥70 years old (*n* = 134), 29.0% were 60–69 years old (*n* = 118), and 23.8% were 50–59 years old (*n* = 97). For the site of onset, the pleura was more commonly involved, observed in 76.9% of cases (*n* = 313), compared to the peritoneum (23.1%, *n* = 94). A total of 47.4% of the participants (*n* = 193) had a history of smoking. A total of 54.1% (*n* = 220) had experienced occupational exposure. The most common years of diagnosis were 2011–2015 with 38.1% (*n* = 155) cases, followed by 37.6% (*n* = 153) in 2006–2010, and 21.6% in 2001–2005. The mean age of diagnosis (±SD) by year was 55.0 ± 15.3 years from 1996–2000, with a tendency to increase, as the following figures demonstrate: 58.8 ± 12.2 years (2001–2005), 62.1 ± 12.9 years (2006–2010), and 65.6 ± 10.6 years (2011–2015; *p* < 0.001; Table 1). 

The proportions of pleural mesothelioma (69.0%) and peritoneal mesothelioma (52.1%; *p =* 0.003) cases in men differed significantly. The mean age (±SD) at the year of diagnosis of pleural mesothelioma (63.1 ± 12.0 years) was higher than the mean age for peritoneal mesothelioma (60.6 ± 12.9 years); however, the difference was not statistically significant (*p =* 0.082). There was no significant difference in terms of the age distribution (*p =* 0637). The smoking status was significantly higher in pleural mesothelioma (57.5%) than in peritoneal mesothelioma (42.6%; *p =* 0.014). The median survival duration (months; 95% CI) from diagnosis to death in pleural mesothelioma was 8.0 (6.2–9.8) months, which was shorter than the 10.0 (3.7–16.3) months recorded for peritoneal mesothelioma (*p* < 0.005, determined by a log-rank test). Survival durations of ≤12 months were more frequent for pleural mesothelioma (59.1%) than for peritoneal mesothelioma (50.0%), and durations ≥60 months were observed in 11.5% of pleural mesothelioma cases, which was less frequent than for peritoneal mesothelioma cases (25.5%). The latency period (years) was investigated in 397 cases, resulting in an average ± SD of 33.3 ± 13.4, with no difference between cases of pleural mesothelioma (*n* = 304; 33.2 ± 13.2) and peritoneal mesothelioma (*n* = 93; 33.5 ± 14.0; Table 2).

The standardized incidence ratio (95% CI) by province of primary exposure from 2009 to 2014, calculated using the 2009 mid-annual population as the standard population, was Chungnam, 3.33 (2.51–4.35); Ulsan, 1.85 (0.97–3.21); Seoul, 1.32 (1.06–1.63); Cheju, 1.24 (0.21–4.10); and Chungbuk, 1.06 (0.56–1.84; Table 3).

## 4. Discussion

In this study, we investigated the characteristics of malignant mesothelioma cases compensated under the asbestos injury relief system in Korea. The findings of previous studies on malignant mesothelioma in Korea are as follows: a total of 39 cases of malignant mesothelioma were approved as occupational diseases from 2011 to 2015. Of these patients, 35 (89.7%) were men and 4 (10.3%) were women. The primary sites of malignant mesothelioma were the pleura, 23 (28.2%); the peritoneum, 11 (28.2%); and the mediastinum, 2 (5.1%), with the data unknown for 3 patients (7.7%) [16]. However, there was a limit to grasping the overall scale, as the annual number of cases was less than 10. According to the malignant mesothelioma cancer registry, 361 men and 195 women were registered from 2009 to 2013, comprising 72 men and 39 women per year [13]. However, there are limitations to epidemiologic studies of malignant mesothelioma caused by asbestos exposure due to a lack of information on occupational or environmental exposure. According to data on deaths, from the WHO database, the number of deaths due to malignant mesothelioma in Korea from 2009 to 2013 was 259 in men and 129 in women, with approximately 52 men and 26 women per year [17]. However, data with low diagnostic validity were also included, and exposure information could not be confirmed. According to the Korean Malignant Mesothelioma Surveillance system, which was active from 2001 to 2012, 171 cases (65%) in men and 91 cases (35%) in women were reported between 2006 and 2010, with about 34 cases were reported in men and 18 cases in women per year [18]. The Malignant Mesothelioma Surveillance system that was created to further these findings has now been discontinued, thus limiting the understanding of the overall scale.

The asbestos injury relief data included cases in which the victim or the bereaved family was voluntarily compensated for being aware of previous exposure to asbestos. In addition, cases detected by actively screening regional environmental health centers for people living in areas with high exposure risk were also included. These data can supplement the insufficient information obtained through interviews with pre-trained asbestos experts along with the existing asbestos exposure data; therefore, it is helpful for studying the epidemiologic characteristics of people exposed to asbestos that develop malignant mesothelioma. However, it is important to note that this study does not represent the incidence of malignant mesothelioma in the entire population of Korea. It covers only 32.0% (178/556) of cases from 2009 to 2013 according to the cancer registry [17].

Korea’s asbestos injury relief system is intended to compensate people who have experienced environmental asbestos injury and occupational asbestos exposure but are not approved for industrial accident compensation. In the case of compensation for occupational exposure, the Industrial Accident Compensation Insurance Act, Public Officials Pension Act, Military Pension Act, Seafarers Act, Fishing Boat Accident Compensation Insurance Act, and Pension for Private School Teachers and Staff Act are excluded from the relief [19,20]. Therefore, a case of occupational exposure that could not be proven by the injured person may be included as an injury relief case. Thus, although “injury relief” aims to compensate for environmental exposure, our research data also included occupational exposure. Malignant mesothelioma caused by exposure to asbestos can be compensated under national occupational disease compensation systems or by civil litigation if occupational or environmental exposure can be clearly demonstrated. However, prompt and accurate compensation may not be achieved in the case of failure to provide proof against the perpetrator or of the disappearance of the perpetrator. The asbestos victim relief schemes were introduced to resolve the issue of victims of asbestos-related diseases not receiving compensation through conventional legal orders. The countries implementing asbestos victim relief schemes worldwide are France (2002), Japan (2006), Belgium (2007), the Netherlands (2007), the United Kingdom (2008), and Korea (the Asbestos Injury Relief System of Korea, 2011) [21].

The findings of our study are as follows: first, the average age at the time of diagnosis of malignant mesothelioma showed a tendency to increase with progress in the reporting years. It has been verified and published by the Tuscan Registry in Italy that aging of the reported cases means that the asbestos exposure has stopped or the intensity has decreased [22]. Our study’s results are also considered to be in line with the decrease in the use of asbestos in Korea [13].

Second, 34.9% of the study participants were women, which was higher than in previous reports. The proportion of women with malignant mesothelioma was 28.4% among cases investigated by ReNaM (Registro Nazionale dei Mesoteliomi), Italy’s malignant mesothelioma surveillance system (1993–2012) [23]. This proportion was 20.6% in Germany’s cancer registry (2009–2013) [24], 22.3% in the United States’ cancer registry and surveillance program (2003–2007) [25], 15.5% in the cancer registry of Australia (1982–2009) [24], and 8.9% in the Dutch registry (2005–2008) [26]. Although the Dutch study was based on asbestos injury relief data, as in our study, the proportion of women in our study was very high. The former Korean surveillance system (which was terminated in 2012) reported a similar pattern between 2001 and 2010, with 33.8% of the cases being female patients [18].

Third, the proportion of peritoneal mesothelioma among all malignant mesotheliomas was 23.1%, which was relatively high compared to other studies (6.5% in Italy [23], 7.5% [24] in Germany, 9.2% [25] in the United States, 5.5% in Australia [26], and 4.2% in the Netherlands [27]). Malignant mesothelioma is a malignant tumor that develops in the mesothelium (the epithelium that lines the thorax or abdomen) when asbestos fibers infiltrate the pleura or peritoneum. It may affect mesothelial cells, such as those of the pleura, peritoneum, pericardium, and tunica vaginalis, but it develops in the pleura in 80–90% of cases and in the peritoneum in 7–10% [28]. Autopsies performed on 1785 patients with malignant mesothelioma in Japan revealed that 68.0% had pleural mesothelioma and 24.1% had peritoneal mesothelioma [29], which is similar to our study. The Korean surveillance system reported a similar pattern with a 66.9% and 27.1% prevalence of pleural and peritoneal mesothelioma, respectively [18].

Fourth, the proportion of men with pleural mesothelioma was 69% and that of women was 31%, which was lower in men and higher in women than reported in other studies. The proportion of men with pleural mesothelioma was 72.5% in Italy [23], 81.4% in Germany [24], 80.1% in the United States [25], and 85.1% in Australia [24]. In this study, the proportion of men with peritoneal mesothelioma was 52.1% and the proportion of women was 47.9%, which was lower than those of men and higher than those of women reported in other studies. The proportion of women with peritoneal mesothelioma was 41.3% in Italy [23], 42.0% in Germany [24], 42.5% in the United States [25], and 26.6% in Australia [26].

Fifth, the proportion of occupational exposure was higher for pleural mesothelioma (57.5%) than for peritoneal mesothelioma (42.6%) in this study. In Italy (1993–2012), the rate of occupational exposure was 54.5% in the pleura and 41.4% in the peritoneum, similar to our study [23]. Considering the proportion of occupational exposure by sex, 71.3% of pleural mesothelioma cases were male patients and 26.8% were female patients, while in peritoneal mesothelioma, 61.2% were male and 22.2% were female. The proportion of occupational exposure among men was high in both types of exposure sites. In an Italian study, the proportion of occupational exposure in the pleura was 65.9% for men and 24.0% for women, whereas in the peritoneum, it was 54.3% for men and 23.0% for women, both of which were high for men and showed a distribution similar to that observed in Korea [23]. The relatively high proportion of peritoneal mesothelioma in women suggests that an exposure other than an occupational one is widely distributed.

Sixth, the median survival duration of peritoneal mesothelioma (10 months) was longer than that of pleural mesothelioma (8 months, *p* < 0.005 determined with a log-rank test). Among population-based studies of more than 300 cases, few studies have investigated the survival duration of pleural and peritoneal mesothelioma simultaneously. In studies in which the median survival of pleural mesothelioma was longer than that of peritoneal mesothelioma, the median survival of pleural mesothelioma was 8 to 10 months and the median survival of peritoneal mesothelioma was 4 to 6 months. These studies were conducted based on malignant mesothelioma registration data and vital statistical data [30,31,32]. Among studies in which the median survival duration of peritoneal mesothelioma was longer than that of pleural mesothelioma, the median survival duration of pleural mesothelioma was reported to be 13 months and that of peritoneal mesothelioma was 20 months according to vital statistical data from Germany [33]. In addition, in a study based on large-scale cohort data in the United States (*n* = 380), the median survival of pleural mesothelioma was 18 months and that of peritoneal mesothelioma was 76 months, which was significantly longer than that of pleural mesothelioma [34]. In the aforementioned study, 83.2% (*n* = 316) of the cases were independent medical evaluations for medical legal purposes. Notably, that study is similar to ours because our study participants had undergone a relief procedure to compensate for malignant mesothelioma. In addition, our study suggests that the proportion of cases of peritoneal mesothelioma, which is relatively easy to treat, was high because it was detected at a relatively early stage.

The mean latent period for pleural/peritoneal mesothelioma in our study (33.2 ± 13.2 and 33.5 ± 14.0 years, respectively) was similar to the values previously reported. A Japanese case analysis study reported a mean latent period of 37.0 ± 13.3 years for malignant mesothelioma, with a latent period of more than 31 years in most cases [35]. Current evidence suggests that the latent period for malignant mesothelioma exceeds 30 years [10,31]; in some studies, the latent period was found to be prolonged to as long as 44.6 years [36]. However, the latency period in a UK cohort of asbestos workers who died of malignant mesothelioma between 1978 and 2015 was relatively short at 22.8 years [37]. The distribution of the latency period in our study was 10–79 years for pleural mesothelioma and 10–75 years for peritoneal mesothelioma, which was consistent with the results of studies suggesting that the latency period may be around 15 years [10].

Seventh, a difference was observed in the distribution of malignant mesothelioma by region. The regions where the incidence was higher than expected were Chungnam (SIR = 3.33; 95% CI = 2.90–4.25), Ulsan (SIR = 1.85; 95% CI = 0.97–3.21), and Seoul (SIR = 1.32; 95% CI = 1.06–1.63). According to a review of environmental asbestos exposure sources in Korea, 29 out of 42 asbestos mines and 16 out of 17 asbestos processing mines are distributed in Chungnam. Ulsan has 34 of 94 chemical complexes nationwide. In addition, 248 out of 412 redevelopment areas are distributed in Seoul [38]. The asbestos injury relief system was created to compensate victims of asbestos-related diseases that were caused by environmental exposure; however, it is also subject to provide compensation if occupational exposure is suspected but not recognized as an occupational disease by the Industrial Accident Insurance System. Therefore, the relationship between the regional distribution of environmental exposure sources and the occurrence of malignant mesothelioma may be somewhat weakened. Nevertheless, regional trends can be used as important information to infer the causes of exposure to asbestos-related diseases. A log-transformed positive correlation between the national cumulative malignant mesothelioma count and cumulative asbestos use was observed in a study from 1994 to 2008 in 56 countries [39]. Our data were five-year observational data, and if the data were accumulated, we could examine the occurrence trends by region.

This study had several limitations. First, it was based on data from the first five years of the enforcement of the Asbestos Injury Relief Act and included diagnoses before the enforcement of the Act. In addition to patients with malignant mesothelioma, bereaved families could also participate in the interview, and recall bias may have occurred during the investigation of exposure history. In addition, the representativeness of the asbestos injury relief data could be somewhat reduced due to the inclusion of cases where contact was lost due to death or other reasons. Also, as mentioned earlier, our data did not represent the incidence of malignant mesothelioma in the entire Korean population. Therefore, the generalization of the interpretation of the results is limited.

Nevertheless, our study had the following strengths: this study is the first epidemiological study of malignant mesothelioma based on asbestos injury relief data in Korea. The diagnoses of malignant mesothelioma by biopsy using immunochemical staining methods showed high medical validity, except for one case diagnosed by computed tomography. This study confirmed the dates of diagnosis and death of malignant mesothelioma cases by checking records and resident registration records. A retrospective cohort can be established if continuous securing of data from the Asbestos Injury Relief Headquarters is made possible, allowing detailed epidemiologic investigations. It has been ten years since the Asbestos Injury Relief system was started. Although the use of asbestos products was banned in Korea in 2009, it is predicted that the number of malignant mesothelioma patients will continue to increase, with an estimated peak in 2038 [13]. Large quantities of data would thus be accumulated in the future that could provide important clues regarding the epidemiologic characteristics of malignant mesothelioma caused by asbestos exposure in Korea. Although there is a limit to revealing the exact epidemiologic characteristics due to the limitation of the representativeness of the data in the current study, it can be used as basic data to help make future policy decisions for malignant mesothelioma.

## 5. Conclusions

The distribution of malignant mesothelioma in Korea identified using the asbestos injury relief system was found to be higher in women and in peritoneal mesothelioma than reported in previous studies. Also, the mean age of diagnosis tended to increase as the diagnosis year progressed. In addition, the survival duration of peritoneal mesothelioma was longer than that of pleural mesothelioma. The regional differences in the occurrence of malignant mesothelioma in Korea identified by the asbestos injury relief data seemed to be related to the size of the asbestos exposure sources, including asbestos mines, asbestos factories, chemical complexes, and redevelopment areas. Although the representativeness of Korea’s asbestos damage relief data is limited, it is possible to assume from them the epidemiologic characteristics of malignant mesothelioma, and the data can help to improve the compensation system and design of future policies.

## Figures and Tables

**Table 1:** General characteristics of study subjects (*n* = 407).

Variables	No. of Subjects (%) or Mean ± SD
Sex	
Male	265 (65.1)
Female	142 (34.9)
Age at diagnosis, years	
Mean ± SD	62.5 ± 12.3
<40	23 (5.7)
40–49	35 (8.6)
50–59	97 (23.8)
60–69	118 (29.0)
≥70	134 (32.9)
Site of onset	
Pleura	313 (76.9)
Peritoneum	94 (23.1)
Smoking history	
Yes	193 (47.4)
No	214 (52.6)
Occupational exposure	
Yes	220 (54.1)
No	187 (45.9)
Age at diagnosis by year ^1^	
1996–2000 (*n* = 11)	55.0 ± 15.3
2001–2005 (*n* = 88)	58.8 ± 12.2
2006–2010 (*n* = 153)	62.1 ± 12.9
2011–2015 (*n* = 155)	65.6 ± 10.6

^1^ *p* < 0.001 determined with t-test by linear regression analysis.

**Table 2:** Comparison of malignant mesothelioma by site of onset (*n* = 407).

Variables	Pleural Mesothelioma (*n*, %)	Peritoneal Mesothelioma (*n*, %)	*p*-Value
Total	313 (100.0)	94 (100.0)	
Sex			
Male	216 (69.0)	49 (52.1)	0.003
Female	97 (31.0)	45 (47.9)	
Age at diagnosis, years			
Mean ± SD	63.1 ± 12.0	60.6 ± 12.9	0.082
<40	17 (5.4)	6 (6.4)	0.637
40–49	25 (8.0)	10 (10.6)	
50–59	71 (22.7)	26 (27.7)	
60–69	92 (29.4)	26 (27.7)	
≥70	108 (34.5)	26 (27.7)	
Smoking history			
Yes	159 (50.8)	34 (36.2)	0.014
No	154 (49.2)	60 (63.8)	
Occupational exposure			
Yes	180 (57.5)	40 (42.6)	0.013
No	133 (42.5)	54 (57.4)	
Survival duration (months)			
Median (95% CI)	8.0 (6.2–9.8)	10.0 (3.7–16.3)	0.005 ^1^
<13	185 (59.1)	47 (50.0)	0.019
13–24	58 (18.5)	12 (12.8)	
25–48	26 (8.3)	8 (8.5)	
49–60	8 (2.6)	3 (3.2)	
>60	36 (11.5)	24 (25.5)	
Latency period (years)			
Mean ± SD	33.2 ± 13.2	33.5 ± 14.0	0.876
10–19	41 (13.1)	13 (13.8)	0.777
20–29	87 (27.8)	26 (27.7)	
30–39	94 (30.0)	31 (33.0)	
40–49	51 (16.3)	11 (11.7)	
≥50	31 (9.9)	12 (12.8)	
Unknown	9 (2.9)	1 (1.1)	

^1^ *p*-value determined with a log-rank test.

**Table 3:** Standardized incidence ratio ^1^ of malignant mesothelioma in Korean provinces, 2005–2014 (*n* = 310).

Province	Frequency of Cases			SIR ^2^		
	Male	Female	Total	Male	Female	Total
Seoul	63	20	83	1.49	1.00	1.32
Gyeonggi	22	15	37	0.50	0.73	0.58
Incheon	9	2	11	0.85	0.39	0.70
Kangwon	3	6	9	0.76	0.88	0.79
Daejeon	5	2	7	1.04	0.73	0.94
Chungnam	32	19	51	2.90	4.25	3.33
Chungbuk	5	6	11	0.67	1.86	1.06
Busan	10	13	23	0.59	1.68	0.93
Ulsan	6	5	11	1.50	2.54	1.85
Daegu	6	2	8	0.57	0.40	0.51
Gyeongnam	15	5	20	1.02	0.73	0.91
Gyeongbuk	5	5	10	0.34	0.80	0.48
Gwangju	4	3	7	0.73	1.28	0.87
Jeonnam	7	3	10	0.61	0.59	0.61
Jeonbuk	6	4	10	0.60	0.94	0.70
Cheju	2	0	2	0.82	0.00	1.24
Total	200	110	310	1.00	1.00	1.00

^1^ Standard population: 2009 regional age-specific Korean population. ^2^ SIR: standardized incidence ratio.

## Data Availability

The data from this study cannot be shared publicly because they contain sensitive patient information and location data. Researchers must inform the Research Ethics Committee of their research purpose and obtain approval for access to the data. For data inquiries about this research, you can contact the administrator of the ethics committee of Soonchunhyang University Cheonan Hospital: schcarib@schmc.ac.kr.
